# Medication-Related Readmissions: Documentation of the Medication Involved and Communication in the Care Continuum

**DOI:** 10.3389/fphar.2022.824892

**Published:** 2022-03-21

**Authors:** Ze-Yun Lee, Elien B. Uitvlugt, Fatma Karapinar-Çarkit

**Affiliations:** Department of Clinical Pharmacy, OLVG, Amsterdam, Netherlands

**Keywords:** hospital readmissions, preventability, medication-related problems, length of stay, quality of heathcare

## Abstract

**Background:** Of all readmissions, 21% are medication-related readmissions (MRRs). However, it is unknown whether MRRs are recognized at the time of readmission and are communicated in the care continuum.

**Objectives:** To identify the prevalence of MRRs that contain a documentation on the medication involved (and therefore are regarded as recognized), and the proportion of communicated MRRs.

**Setting**: The study was performed in a teaching hospital.

**Methods:** In a previous study, a multidisciplinary team of physicians and pharmacists assessed the medication-relatedness, the medication involved and preventability of unplanned readmissions from seven departments. In the current cross-sectional study, two pharmacy team members evaluated the patient records independently. An MRR was regarded as recognized when the medication involved was documented in patient records. An MRR was regarded as communicated to the patient and/or the next healthcare provider when the medication involved or a description was mentioned in discharge letters or discharge prescriptions. The relationship between documented MRRs and whether the MRR was preventable as well as the relationship between (un)documented MRRs and the length of stay (LOS) were assessed. Descriptive data analysis was used.

**Results:** Of 181 included MRRs, 72 (40%) were deemed preventable by the multidisciplinary team. For 159 of 181 MRRs (88%), a documentation on the medication involved was present. Of 159 documented MRRs, 93 (58%) were communicated to patients and/or caregivers, 137 (86%) to the general practitioner, and 4 (3%) to the community pharmacy. The medication involved was documented less often for potentially preventable MRRs than for non-preventable MRRs (78 vs. 95%; *p* = 0.002). The LOS was longer for MRRs where the medication involved was undocumented (median 8 vs. 5 days; *p* = 0.062).

**Conclusion:** The results of this study imply that MRRs are not always recognized, which could impact patients’ well-being. In this study an increased LOS was observed with unrecognized MRRs. Communication of MRRs to the patients and/or the next healthcare providers should be improved.

## Introduction

Unplanned readmissions are an indicator of patient safety. Approximately 20% of patients in the United States experience unplanned readmissions within 30 days after discharge (Jencks et al., 2009). These readmissions impact both patients and healthcare providers since they are associated with an increase in morbidity, mortality, health expenditures, and increased workload and stress for healthcare providers (Carter et al., 2018; Upadhyay et al., 2019; Shaw et al., 2020).

A previous study shows that medication is a major cause of potentially preventable readmissions (van der Does et al., 2020). A systematic review reported that 21% of readmissions were medication-related, and 69% of these were potentially preventable (El Morabet et al., 2018). A recent Dutch study with 1,111 readmissions showed that 16% of readmissions were medication-related, of which 40% were potentially preventable (Uitvlugt et al., 2021). Polypharmacy, medication changes during index admission (IA) and hospitalizations before IA could increase the risk of an MRR for elderly patients (Uitvlugt et al., 2021). Other studies reported that harm from medicine could significantly increase the length of stay (LOS) (Sánchez Muñoz et al., 2007; Davies et al., 2009; Sandoval et al., 2020; Uitvlugt et al., 2021).

To adequately manage medication-related readmissions (MRRs) and minimize extension in the LOS, healthcare providers must recognize the medication involved. In addition, information about the medication involved must be transferred in the care continuum to prevent re-prescription of medication that causes hospitalization. This could contribute to adequate follow-up by primary healthcare providers for patients who for example need help in medication self-management (van der Linden et al., 2006; Auerbach et al., 2016). Patients must also be informed about the impact of their medication use to prevent unintentional re-use of harmful medication (Yam et al., 2010; Auerbach et al., 2016).

Previous studies focusing on the recognition of medication-related admissions showed that these were not always recognized by healthcare providers. Non-adherence, prescribing errors (e.g., unintended omission of medication) and certain side effects that overlap with symptoms of co-morbidities were missed (Sánchez Muñoz et al., 2007; Witherington et al., 2008; Riordan et al., 2016; Roughead et al., 2016; Huang et al., 2020; de Lemos et al., 2021). Furthermore, a descriptive study showed that of 104 adverse drug reactions patients experienced during hospitalization only 51% were documented in discharge letters for the general practitioner (van der Linden et al., 2006). However, these previous studies did not focus on the recognition of (potentially preventable) MRRs and their communication to patients and/or the next healthcare providers.

Therefore, the objectives of this study were to: 1) identify the prevalence of documented (and thus recognized) MRRs at the time of readmission, 2) examine the relationship between documented MRRs and whether the MRR was preventable, 3) examine the relationship between documented MRRs and the LOS, and 4) assess the proportion of MRRs that were communicated to patients or caregivers, and/or the next healthcare providers.

## Materials and Methods

### Study Design and Participants

This cross-sectional single-center observational study was conducted at the OLVG teaching hospital in Amsterdam, the Netherlands. Data were derived from prior research that focused on all causes of readmissions at seven participating departments (van der Does et al., 2020; Meurs et al., 2021). Patients were prospectively included if they were readmitted between July 2016 and February 2018. Methodological approval was obtained from the scientific review committee of the hospital (*Advies Commissie Wetenschappelijk Onderzoek Medische Ethische Commissie*, ACWO-MEC; registration number 16-028). Patient data were acquired and managed as compliant with the privacy regulations.

This study used definitions, inclusion and exclusion criteria similar to that of the prior research (van der Does et al., 2020; Meurs et al., 2021). An index admission (IA) was defined as the first admission within the study period of July 2016 and February 2018 where a patient was included. Inclusion criteria were unplanned readmissions within 30 days after discharge from an IA to one of the seven participating clinical departments, namely cardiology, gastroenterology, internal medicine, neurology, psychiatry, pulmonology and general surgery. These departments were selected owing to their readmission rates in previous years in OLVG. Only adult patients (≥18 years) were included. Exclusion criteria were patients transferred to another hospital during IA, patients who left against medical advice during IA, readmissions due to attempted suicide and readmissions unrelated to IA. For the current study, readmissions regarded as non-medication-related were also excluded (see the next paragraph). If a patient had multiple readmissions during the study period, each readmission was assessed separately. This meant that for a second readmission the first readmission was regarded as an IA to assess the causality and preventability of the second readmission in relation to the first readmission.

### Usual Care

Medication monitoring was performed by pharmacists using a computerized system to check for the right dose or medication interactions. During the study, transitional care was present at some departments and was implemented further gradually. This included medication reconciliation at hospital admission and discharge. No formal medication review was performed but obvious errors were addressed (e.g., lack of a laxative when an opioid is prescribed or no indication for hypnotics upon discharge). A pharmacy team member explained medication changes to the patient and communicated these to the next healthcare providers. Further details are described in the main study (Uitvlugt et al. 2021).

### Causality and Preventability Assessments: The Gold Standard

**FIGURE 1:**
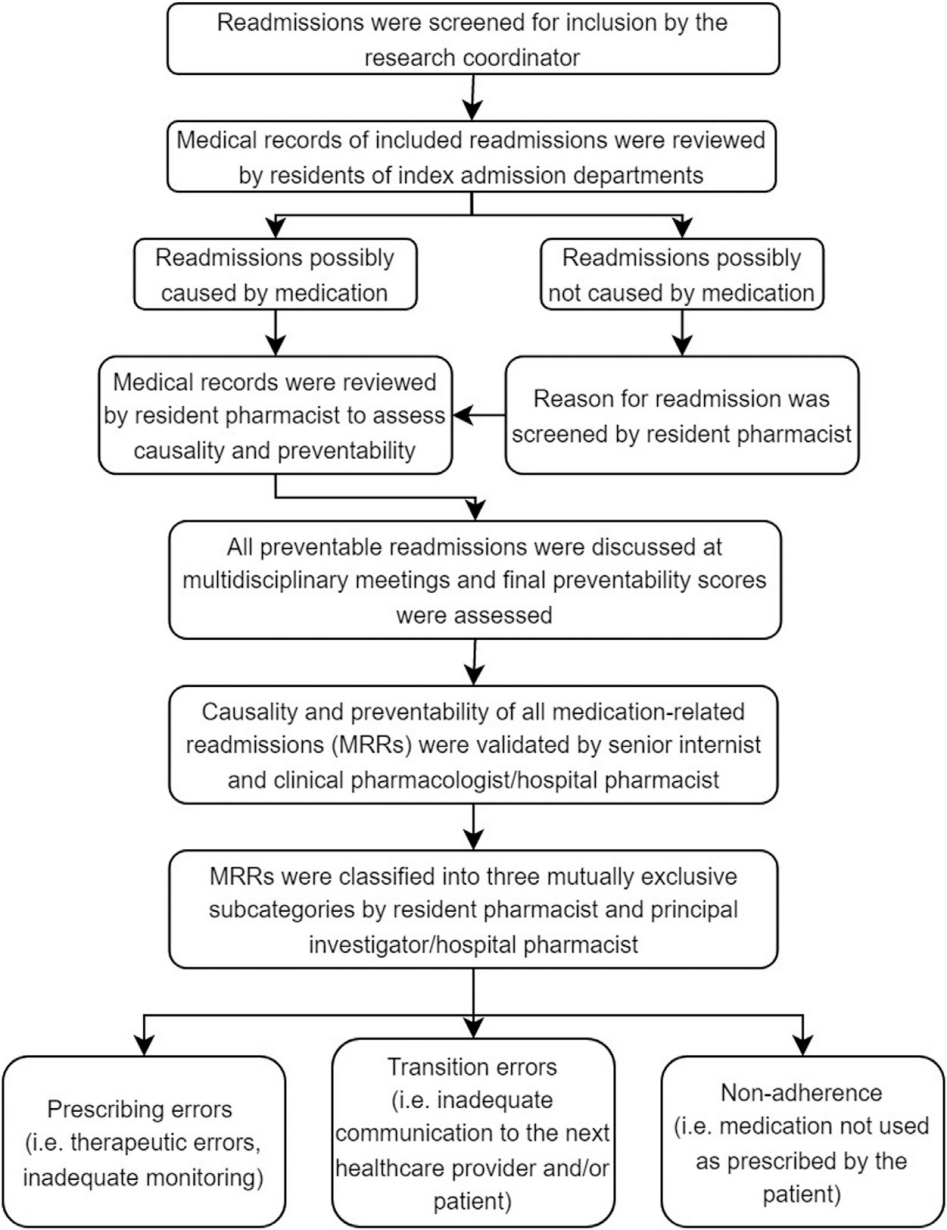
Process of causality and preventability assessments of readmissions (Uitvlugt et al., 2021; van den Bemt and Egberts 2007; van der Does et al., 2020; Warlé-van Herwaarden et al., 2015). Prescribing errors were defined as medication errors, among others, dosing errors, contra-indications, drug interactions, omission errors or overdosing. Non-adherence was defined as a refill rate lower than 0.8. The refill rate was calculated by dividing the number of daily doses dispensed with the total number of days between the first and last prescription in a period of 8 months before readmission. An admitting medical doctor assessed therapy adherence if the refill rate could not be calculated. If the admitting doctor mentioned a patient of being non-adherent, the patient was classified as such.

Previous studies have published the methodology for assessing the medication-relatedness and preventability of readmissions more extensively, see Figure 1 for a summary of the different steps (van der Does et al. 2020; Uitvlugt et al. 2021). Initially, a research coordinator (medical doctor) screened all readmissions to determine whether they met inclusion criteria. Afterwards, each resident of the seven IA departments and a resident of the hospital pharmacy double-checked the cases to identify possible MRRs. A distinction was made between readmissions caused by medication and readmissions with other causes apart from medication (e.g. diagnostic, management, surgical causes).

Questionnaires were used for the assessments (see Uitvlugt et al., 2021, Supplementary material of reference, Table 2)**.** Causality (i.e., relatedness of the suspected medicine(s) to the readmission) was assessed, and readmissions were classified as possible causal (total score: + 4), probable causal (total score: 0–3) or unlikely causal (total score: < 0). Then, possible and probable causal readmissions were categorized as MRRs and the preventability of those MRRs was assessed using the algorithm by Schumock and Thornton which has been used in many studies and systematic reviews (El Morabet et al., 2018; Uitvlugt et al., 2021). A scoring scale of 1–6 was used to classify the preventability of a readmission. A readmission was deemed potentially preventable (total score ≥4) if it was caused by a medication error (e.g., therapeutic errors, dosing errors, inadequate monitoring, incorrect use, lack of preventative measure). All potentially preventable readmissions were discussed at multidisciplinary meetings attended by all residents of the participating departments, including hospital pharmacists to reach consensus. A final preventability score was assessed.

Moreover, a senior internist and hospital pharmacist validated all MRRs, as identified at the multidisciplinary meetings. Finally, potentially preventable MRRs were classified into three mutually exclusive subcategories: prescribing errors, transition errors or non-adherence (see Figure 1 for the definitions). This final validation of MRRs was considered the gold standard.

### Data Collection and Classification

A research coordinator collected baseline data including patients’ demographics, medical history, IA and readmission characteristics. A resident of the hospital pharmacy and a senior hospital pharmacist visually checked the data on extreme values and missing data (with histograms and scatterplots). Also, as an additional validity check, manually collected data were compared with data extractions from the hospital information system (EPIC Hyperspace, Verona, United States). Differences were discussed, and consensus was met. If consensus could not be met a senior researcher made the final decision.

Data regarding the documentation and communication of medication involved in the readmission were extracted from the hospital information system (see below). Two pharmacy team members evaluated the patient records independently A senior researcher (hospital pharmacist) double-checked all data and consensus was met.

### Documentation of Potentially Preventable and Non-Preventable Medication-Related Readmissions

The patient’s readmission records were screened to assess whether and when an MRR was documented and by whom (e.g., clinical notes of physicians, nurses, pharmacists, discharge letters and/or discharge prescriptions). In addition, documentation of the preventability was assessed if applicable. An MRR was regarded as documented—and therefore recognized as medication-related by healthcare providers—when the medication involved was mentioned in the patient records. The record should at least contain the name of the medicine or the medication group (e.g., antihypertensive medication or diabetes medication) as multiple medications could result in a readmission. For preventability, documentation in the patient records should contain the potentially preventable cause for the readmission (e.g., undertreatment, non-adherence, medication error).

MRRs were classified as documented ≤ 24 h of the readmission, > 24 h following readmission or not documented. This last group consisted of readmissions that were classified as MRRs after the causality and preventability assessments of the senior internist and hospital pharmacist (the gold standard).

### Communication of Potentially Preventable and Non-preventable Medication-Related Readmissions

The patient records were screened to assess whether MRRs were communicated to patients or caregivers, and/or the next healthcare providers (e.g., general practitioner and community pharmacy). An MRR was regarded as communicated to the general practitioner when the discharge letter specified medication as the cause of the readmission, or when the clinical notes specified a phone call to the general practitioner regarding the MRR. For the community pharmacy, discharge prescriptions were checked to assess whether they contained information on medication-relatedness. Preventability of an MRR was regarded as communicated if specification regarding the potentially preventable cause of the readmission was recorded (e.g., patient non-adherence, medication error). Finally, the patient records were checked to assess whether there was documentation about informing the patient and/or their caregivers regarding the medication-relatedness and potential preventability of their readmission.

### Main Outcome Measures

The primary outcome was the prevalence of MRRs that contained documentation on the medication involved in the patient records at the time of readmission. Secondary outcomes were the relationship between documented MRRs and whether the MRR was preventable as well as the relationship between (un)documented MRRs and the LOS. Lastly, the proportion of communicated MRRs to patients or caregivers, and/or the next healthcare providers was assessed.

### Data Analysis

All analyses were performed using SPSS version 22.0 (IBM SPSS, Chicago, IL, United States). Categorical variables were presented as percentages. Normally distributed variables were presented as means and standard deviation (SD). Non-normally distributed continuous variables were presented as medians and interquartile range (IQR). Baseline characteristics of potentially preventable and non-preventable MRRs were compared using the independent *t*-test for continuous variables and the Pearson Chi-square test for frequencies. Descriptive data analysis was used to assess the proportion of readmissions that were documented and communicated. The Pearson Chi-square test was performed to examine the relationship between documented MRRs and whether the MRR was preventable. In addition, the Mann-Whitney *U* test was performed to examine the relationship between documented MRRs and the LOS. A *p*-value < 0.05 was considered statistically significant.

## Results

**FIGURE 2:**
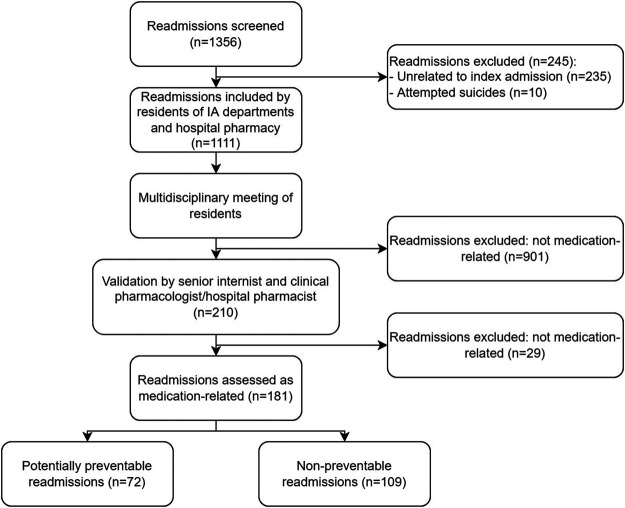
Patient selection for the current study.

A total of 1,356 readmissions ≤30 days after discharge from IA were screened. From this number, 245 were excluded, see Figure 2. Of the remaining 1,111 readmissions (for 873 unique patients), 210 were initially classified as medication-related by residents. After the validation by the senior internist and hospital pharmacist, 181 (16%) readmissions were assessed as MRRs and were included in this study.

Of these 181 MRRs, 72 (40%) were assessed as potentially preventable, and 109 (60%) were assessed as non-preventable (Figure 2). Prescribing errors (35%) and non-adherence (35%) were the most common causes of potentially preventable MRRs. However, 30% was caused by transition errors. Table 1 shows the baselines and readmission characteristics of the included patients. The mean age of patients with a potentially preventable MRR was 69.5 years (SD 13.7), and that of non-preventable MRRs was 68 years (SD 13.8). Gender was equally represented for potentially preventable and non-preventable MRRs. There were no significant differences between (sub) groups.

**TABLE 1:** Baseline characteristics of potentially preventable and non-preventable medication-related readmissions (MRRs) included in this study.

Characteristics^a^	Potentially preventable MRRs (*n* = 72)	Non-preventable MRRs (*n* = 109)
**Patient characteristics**		
Age, mean (SD)	69.5 (13.7)	68 (13.8)
Gender, male, *n* (%)	38 (52.8)	63 (57.8)
Language barrier present, *n* (%)	25 (34.7)	32 (29.4)
Cognitive impairment, *n* (%)	26 (36.1)	21 (19.3)
Living situation, *n* (%)		
• Together with partner/family	35 (48.6)	63 (57.8)
• Alone	25 (34.7)	33 (30.3)
• Institution	10 (13.8)	13 (11.9)
**Medical history**		
CCI score, median (IQR)	1 (0–3)	2 (1–3)
eGFR < 50 ml/min/1.73m^2^, *n* (%)	24 (33.3)	29 (26.6)
Previous hospital admission (< 6 months), *n* (%)	37 (51.4)	58 (53.2)
Previous ED visit (< 6 months), *n* (%)	22 (30.6)	24 (22.0)
**IA characteristics**		
Unplanned admissions, *n* (%)	60 (83.3)	77 (70.6)
Length of stay in days, median (IQR)	7 (3–13)	4 (2–10)
Departments, *n* (%)		
• Cardiology	17 (23.6)	14 (12.8)
• Gastroenterology	13 (18.0)	6 (5.5)
• Internal medicine	17 (23.6)	52 (47.7)
• Neurology	2 (2.8)	1 (0.9)
• Psychiatry	0	1 (0.9)
• Pulmonology	11 (15.3)	25 (22.9)
• General surgery	12 (16.7)	10 (9.2)
Discharge to home, *n* (%)	62 (86.1)	96 (88.1)
Discharge letters sent to GP, *n* (%)	61 (84.7)	92 (84.4)
Number of medication at discharge, mean (SD)	12.6 (5.4)	10.1 (4.6)
Number of medication changes, median (IQR)	3 (2–6)	3 (1–5)
**Readmission characteristics**		
Time between IA and readmission in days, median (IQR)	10.5 (4.3–18.9)	8 (5–15)
Early readmission (≤ 7 days), *n* (%)	27 (37.5)	53 (48.6)
Length of stay in days, median (IQR)	6 (3–11)	5 (2–7.5)
Departments, *n* (%)		
• Cardiology	19 (26.4)	16 (14.7)
• Gastroenterology	10 (13.9)	9 (8.3)
• Internal medicine	20 (27.8)	49 (45.0)
• Neurology	4 (5.6)	2 (1.8)
• Psychiatry	0	1 (0.9)
• Pulmonology	9 (12.5)	22 (20.2)
• General surgery	8 (11.1)	8 (7.3)
• ICU	2 (2.8)	2 (1.8)

a There were no statistically significant differences between groups. CCI , Charlson Comorbidity index; eGFR , estimated glomerular filtration rate; ED , emergency department; IA = index admission; GP , general practitioner; ICU , intensive care unit.

### Documentation of Potentially Preventable and Non-preventable Medication-Related Readmissions

The medication involved was documented in the patient records for 159 of 181 (88%) MRRs, in which 152 (84%) were documented ≤ 24 h of the readmission (see Table 2. The preventability was documented for 51 of 72 (71%) potentially preventable MRRs. The MRRs were often first documented by either a physician (75%) or a nurse (12%).

**TABLE 2:** Documentation of potentially preventable and non-preventable medication-related readmissions (MRRs) in patient records.

Documentation of MRRs	Total MRRs (*n* = 181)	Potentially preventable (*n* = 72)	Non-preventable (*n* = 109)
Medication-relatedness documented, *n* (%)^a^	159 (87.8)	56 (77.8)^a^	103 (94.5)^a^
• Documented ≤24 h, *n* (%)	152 (84.0)	52 (72.2)	100 (91.7)
• Preventability documented, *n* (%)	NA	51 (70.8)	NA
• Documented first by			
o physician, *n* (%)	136 (75.1)	51 (70.8)	85 (78.0)
o nurse, *n* (%)	22 (12.2)	4 (5.6)	18 (16.5)
o hospital pharmacist, *n* (%)	1 (0.6)	1 (1.4)	0

a Difference in proportions documented between potentially preventable MRRs, and non-preventable MRRs, was statistically significant (*p* = 0.002). *NA, not applicable*.

The medication involved documented for non-preventable MRRs was more than that of the potentially preventable MRRs (95 versus 78%; *p* = 0.002, see Table 2).

### Impact on Length of Stay

Table 3 shows the median LOS for MRRs documenting the medication involved versus MRRs without any documentation. Generally, the median LOS of all undocumented MRRs was longer than that of MRRs that documented the medication involved (median 8 vs. 5 days; *p* = 0.062). Similar results were found for the subgroups of non-preventable and potentially preventable MRRs.

**TABLE 3:** Differences in length of stay (LOS) between potentially preventable and non-preventable medication-related readmissions documented and those not documented during readmission.

Medication-related readmissions (*n* = total number; documented)	LOS documented, median in days (IQR)	LOS undocumented, median in days (IQR)	*p*-value^*^
**All (n = 181;159)**	5 (2–8)	8 (3.8–12.3)	*p* = 0.062
**Non-preventable (n = 109;103)**	5 (2–7)	8 (2–9)	*p* = 0.51
**Potentially preventable (n = 72;56)**	5 (2–11)	8 (4.3–13)	*p* = 0.12
• Prescribing errors (*n* = 25; 18)	4 (1–8.8)	7 (3–13)	*p* = 0.42
• Non-adherence (*n* = 25; 20)	5 (2–15.3)	5 (4–11)	*p* = 0.82
• Transition errors (*n* = 22; 18)	6 (3–9)	13 (11–39.5)	*p* = 0.010^*^

* Difference in LOS between documented preventable medication-related readmissions, and undocumented preventable medication-related readmissions, caused by transition errors was statistically significant.

The bold values represent the main groups: non-preventable and preventable MRRS.

MRRs caused by transition errors had a significantly longer LOS when the medication involved was undocumented (median 13 days undocumented vs. 6 days documented; *p* = 0.010).

### Communication of Potentially Preventable and Non-preventable Medication-Related Readmissions

Table 4 shows the proportion of MRRs communicated to patients or caregivers, and/or the next healthcare providers. No documentation on the medication involved was recorded for 22 of 181 MRRs (12%). These MRRs were not communicated to patients or caregivers, and/or the next healthcare providers.

**TABLE 4:** Communication of documented potentially preventable and non-preventable medication-related readmissions (MRRs) to patients or caregivers, and/or the next healthcare providers.

Communication of documented MRRs	Total documented MRRs (*n* = 159^a^)	Documented potentially preventable MRRs (*n* = 56)	Documented non-preventable MRRs (*n* = 103)
General practitioner, n (%)	137 (86.2)	47 (83.9)	90 (87.4)
• No discharge letter sent, n (%)	12 (8.8)	5 (8.9)	7 (6.8)
Community pharmacy, n (%)	4 (2.5)	1 (1.8)	3 (2.9)
Patients and/or caregivers, n (%)	93 (58.5)	35 (62.5)	58 (56.3)

a Of 181 MRRs, 159 contained a documentation on the medication involved. For 22 MRRs (12%) there was no documentation on the medication involved resulting in an MRR., For these 22 MRRs, there was also a lack of communication to patients or caregivers, and/or the next healthcare provider(s).

Of 159 MRRs that had documentation on the medication involved, 137 (86%) were communicated to the general practitioner. The information was specified in different sections of the discharge letter, e.g., the anamnesis, the patient evaluation section and/or in the conclusion. Four of 159 MRRs (3%) were communicated to the community pharmacy. For 93 MRRs (58%), information was found in patient records regarding informing the patients and/or their caregivers about the medication-relatedness of the readmission.

## Discussion

Our study showed that for 88% of MRRs, the medication involved was documented in the patient records. The medication involved was lacking more often for potentially preventable MRRs than for non-preventable MRRs. An increase in LOS was also found for unrecognized MRRs.

The proportion of recognized MRRs (88%) in this study is higher than those found in previous studies with frequencies of 4.01 and 51.2% (Hohl et al., 2005; Sánchez Muñoz et al., 2007). This discrepancy could be attributed to the inconsistency of study methods (e.g., assessing all medication-related causes vs. only adverse drug events), study population (e.g., all adult patients vs. only elderly patients) and recognition assessment (e.g., manually vs. scoring system). Also, medication reconciliation performed in several departments could aid in the recognition of MRRs in this study. Additionally, previous studies focused on the recognition of adverse drug events of medication-related admissions (Hohl et al., 2005; Sánchez Muñoz et al., 2007). In the current study, patients were those with possible multiple (re)admissions, which could aid the recognition of the readmissions and higher documentation rates. None of the previous studies focused on the recognition of potentially preventable and non-preventable MRRs, and therefore no direct comparison could be made. Potentially preventable readmissions occurred more often at a different department than where the IA occurred. Thus, the readmission was due to a new problem requiring a new medical specialty, which could make it more difficult to recognize these readmissions (Meurs et al., 2021).

Our findings showed that 86% of MRRs are communicated to the GP, which is higher than the 51% reported by a previous study (van der Linden et al., 2006). This variability in prevalence rates could be explained by a difference in assessment methods. In our study we focused on whether the medicine or medication group involved in the patient’s readmission was communicated to the GP, whereas in the previous study the communication of adverse drug reactions was assessed. Community pharmacists were not informed on MRRs which is in line with a previous study (van der Linden et al., 2006). However, it is important to inform community pharmacies as well as this can improve medication surveillance.

We found evidence that communication of the medication-relatedness of the readmission was present in 58% of readmissions. A previous study showed that patients’ and healthcare providers’ perspectives on the medication-relatedness of readmissions and their preventability differed (Uitvlugt et al., 2020). Consensus between patients and healthcare providers with respect to the role of medication is necessary to achieve optimal pharmacotherapy outcomes. Patients need to be aware of the reason of their readmission, also to prevent future MRRs with the same medication. In a recent study patients reported that they would like to receive information on medication management or adverse effects both verbally and written (Daliri et al., 2019).

### Study Limitations

A thorough search of the literature reveals that this is the first study to assess the recognition of preventable MRRs and their communication to patients or caregivers, and/or the next healthcare providers. However, some limitations must be considered when interpreting the findings. First, the external generalizability might be limited as this study was performed in a single hospital with a selected number of clinical departments. By including high-risk departments, data assessments efficiency was improved since reviewing data of all readmitted patients is time-intensive. Second, MRRs were assessed by using information from patient records, thus, relevant information from patients and healthcare providers not documented in the patient records could have been missed. For example, communication to patients and/or their caregivers may occur without any documentation. However, we assessed clinical notes that were present from multiple healthcare providers (e.g. nurses, physicians, pharmacists) and not solely discharge letters to minimize the chances of missing any documentation. Third, multidisciplinary meetings might have been held during a patient’s readmission, aiding in the recognition and documentation of MRRs by healthcare providers. This could result in an overestimation of recognized cases. However, the results were expected to be minimally affected as most patients were already discharged when the multidisciplinary evaluations took place. Fourth, this study was exposed to the subjectivity of the researcher who assessed the cases, which could lead to bias. However, a pharmacy team member double-checked the assessments and consensus was met by consulting a senior researcher, which increased the reliability of the results. Finally, this study was underpowered to assess significant differences between study (sub) groups, including LOS, age or type of medication due to the small study population. The results on LOS could be biased and should be interpreted with caution as no adjustments on confounders were performed due to the small number of patients. Further multicenter research with a larger patient population is required to confirm the findings and increase generalizability.

### Implications for Clinical Practice

The findings of this study suggest considerable scope for improvements in the clinical healthcare practice and patients’ outcomes. First, cooperation between healthcare providers and patients may improve recognition of MRRs. Uitvlugt *et al.* showed that patients could offer more medication-related information (e.g., non-adherence) regarding the period after discharge than healthcare providers because this information is often not documented in the patient records (Uitvlugt et al., 2020). Therefore, a patient interview regarding the reason of readmission should be implemented to improve recognition of an MRR. Second, to minimize loss of information during transfers in the care continuum, medication-related information should be reported in a standardized method in discharge letters and discharge prescriptions. De Lemos *et al.* developed a reporting tool to automatically generate a letter to community healthcare providers after medication-related causes and required (follow-up) actions are entered (de Lemos et al., 2021). Lastly, healthcare providers should be trained to recognize potentially preventable MRRs and extract information of patients who are at risk of potentially preventable MRRs so that preventative measures could be implemented. This requires hospitals to systematically evaluate the preventability of readmissions. Also, by encouraging the use of medication error reporting systems more insight could be gained into the extent and impact of medication-related harm to reduce MRRs. Previous studies showed a high proportion of underreporting of medication errors (approximately 50–60%) among healthcare providers (Jember et al., 2018; Mutair et al., 2021). However, Elden *et al.* found a decrease of approximately 50% of medication errors (from 6.7 to 3.6%, *p* ≤ 0.001) through application of error reporting among healthcare providers (Elden and Ismail, 2016). A systematic approach to analyze, monitor and recognize medication errors and MRRs could improve quality of healthcare and patient safety. Also, automatic systems could help in recognizing potential MRRs and future studies should evaluate the use of artificial intelligence or algorithms to detect MRRs.

## Conclusion

This study demonstrated that the medication involved was documented in the patient records for 88% of MRRs. The medication involved was lacking more often for potentially preventable MRRs. These findings imply that MRRs are not always recognized, which could impact patients’ well-being. An increased LOS was observed in this study for MRRs that were unrecognized, and a lack of communication in the care continuum was identified. Further multicenter research with a larger patient population is required to confirm the findings of this study.

## Data Availability

The original contributions presented in the study are included in the article/Supplementary Material, further inquiries can be directed to the corresponding author.
